# The influence of parents and the home environment on preschoolers' physical activity behaviours: A qualitative investigation of childcare providers' perspectives

**DOI:** 10.1186/1471-2458-11-168

**Published:** 2011-03-17

**Authors:** Patricia Tucker, Melissa M van Zandvoort, Shauna M Burke, Jennifer D Irwin

**Affiliations:** 1 School of Occupational Therapy, 1201 Western Road, Elborn College, University of Western Ontario, London, ON, N6G 1H1, Canada; 2 Middlesex-London Health Unit, 50 King St., London, ON, N6A 5L7, Canada; 3 School of Health Studies, Arthur & Sonia Labatt Health Sciences Building, University of Western Ontario, London, ON, N6A 5B9, Canada

**Keywords:** physical activity, preschoolers, home environment, parents, childcare providers

## Abstract

**Background:**

Physical activity offers numerous physiological and psychological benefits for young children; however, many preschool-aged children are not engaging in sufficient activity. The home environment, inclusive of parent role modeling, has been identified as influencing preschoolers' physical activity. This study sought to examine childcare providers' perspectives of the importance of parents and the home environment for supporting the physical activity behaviours of preschool-aged children (aged 2.5-5 years) attending childcare.

**Methods:**

A heterogeneous sample of childcare providers (*n *= 84; response rate 39%) working at childcare facilities in London, Ontario participated. Thirteen semi-structured focus groups were conducted in London centres between February 2009 and February 2010. Focus groups were audio recorded and transcribed verbatim and inductive content analysis was used to code and classify themes. A number of strategies were used to verify the trustworthiness of the data.

**Results:**

Childcare providers acknowledged their reliance on parents/guardians to create a home environment that complements the positive physical activity messaging children may receive in childcare. Moreover, childcare staff highlighted the need for positive parent role modeling and parent support to encourage active healthy lifestyles among young children.

**Conclusion:**

This study's findings highlight the need for increased parent-caregiver partnering in terms of communication and cooperation in service of promoting appropriate amounts of physical activity among London preschoolers.

## Background

Among Canadian preschoolers, obesity rates are disturbing; research suggests that between 26 and 30% of young children are overweight or obese [1,2]. The high rate of childhood obesity is disconcerting as obesity has been linked to type 2 diabetes, hypertension, hyperlipidemia, sleep apnea, and psychological issues [3,4]. The increased incidence is also problematic because individuals who are overweight or obese during childhood are more likely to be obese as adults [5]. Specifically, researchers estimate that overweight preschoolers are four times more likely to be overweight as adults [6].

Physical activity is an important behaviour for young children to adopt as it provides many physiological and psychological health benefits, including preventing obesity [4]. However, within Canada, the National Longitudinal Survey of Children and Youth indicates that only 36% of 2-3 year olds and 44% of 4-5 year olds participate in sport and physical activity each week [7]. Tucker and Irwin reported a slightly higher participation rate [8], with 55% of London Ontario preschoolers engaging in 60 minutes of physical activity each day. Tucker and Irwin's participation rates are similar to those found among preschoolers internationally. In a systematic review of 39 international studies, Tucker reported that 54% of preschoolers engaged in 60 minutes of physical activity per day [9]. If "sufficient physical activity" was defined as aligning with the National Association for Sport and Physical Education (NASPE) Guideline (which recommends 60 minutes of *structured *and a minimum of 60 minutes of *unstructured *daily physical activity for the preschooler cohort), only 23% would have engaged in appropriate amounts of activity [9]. It is clear that many Canadian and international preschoolers are not engaging in appropriate amounts of physical activity. Given that activity levels have been found to decline with age [10], and that the preschool years may be a transitional time for children moving from childcare to the school system, it is imperative that physical activity be encouraged and active lifestyles fostered among this young population.

Children's health behaviours are formed at an early age [11,12], and many of these behaviours are largely under the influence of their parents. In fact, the family unit is particularly important for the development of young children's activity-related attitudes, beliefs, preferences, and behaviours [13,14]. In addition to parental control over children's behaviours, Sallis and colleagues purported that family-related variables correlate strongly with children's physical activity habits [15]. Specifically, physical activity participation among preschool-aged children is strongly associated with parental physical activity behaviours and prompts from adults [15-18]. Moreover, the home environment itself (e.g., rules regarding activity, accessibility of play spaces, etc.) has also been acknowledged as impacting physical activity participation among young children, accounting for 16% of the variance in physical activity levels[18]. Therefore, parental role modeling and the home environment are important influences on the physical activity behaviours of preschool-aged children.

The present study took place within the context of a larger program of research. The goal of this research program was to gain an understanding of childcare providers' perspectives of: preschoolers' current physical activity behaviours; the barriers and facilitators to physical activity participation at childcare facilities; and suggestions for improving physical activity opportunities in childcare centres [19,20]. This research was undertaken because of the strong emphasis and increased attention placed on the childcare setting as an important venue for supporting physical activity participation among children [21,22]. Despite the strong research focus on physical activity participation within the childcare setting, childcare providers emphasized the influence that parents and the child's home environment has on the physical activity behaviours of preschoolers attending childcare. These insightful and relatively under-examined issues (related to the role of parents and the home environment on preschooler's physical activity) represent the focus of this current paper.

Parents of preschoolers have previously acknowledged their dependence on childcare staff to ensure their children are sufficiently active [23,24]; however, the perspectives of childcare staff with regard to the role of parents in increasing children's physical activity has not been examined. An understanding and acknowledgement of childcare providers' expectations and perceptions is important for caregivers and health professionals given the substantial amount of time children spend in daycare and the pivotal role childcare providers play in shaping preschooler's behaviour. Therefore, the purpose of this study was to examine childcare providers' perspectives of the importance of parents and the home environment for supporting physical activity behaviours of preschool-aged children (aged 2.5-5 years) attending childcare.

## Methods

### Recruitment

In total, 13 semi-structured focus groups were conducted at public childcare centres in London, Ontario (from diverse areas of the city) between February 2009 and February 2010 to address the purposes of the larger study. The first eight focus groups were conducted between February and March 2009 with providers from one public childcare organization. An additional five focus groups were conducted after an Advocacy Development Grant from the Heart and Stroke Foundation of Ontario was awarded to expand the study. These focus groups were undertaken in January and February of 2010 with childcare providers from an additional two public childcare organizations. Participating childcare staff were drawn from three organizations that ranged in the number of facilities within the city (i.e., 1, 12, and 13 centres across London). The additional focus groups provided a more in-depth understanding of the importance childcare providers placed on the home environment and the parent as an equal partner for supporting and encouraging physical activity participation among preschoolers. This theme was also discussed in the first round of focus groups, but was not captured in the previously published articles [19,20].

### Participants

A total of 84 childcare providers (mean age = 33 years; 99% female; mean length of experience = 9 years) from 16 childcare centres in London, Ontario participated in this study. Eighty-one percent of participants indicated that they were full-time childcare providers, and the remaining 19% indicated that they were either part-time or casual (e.g., occasional employment) childcare providers. The majority of participants (79%) had a college education, and an additional 13% had either a university or post-graduate degree. While the ethnicity of the childcare providers varied, the majority (87%) who participated were Caucasian.

### Focus Groups

All of the focus groups took place at the childcare centres that employed the majority of participants. Four of the focus groups took place during childcare hours and nine were held in the evening when the centres were closed. One of the focus groups took place at a single-site organization, while the other 12 were held at branches of two different public childcare organizations. Four to 10 childcare providers participated in each of the focus groups. In total, 84 childcare providers (of a possible 214) from 16 childcare centres participated in one of the 13 focus groups for a response rate of 39%.

The focus groups each lasted between 1 and 1.5 hours and were facilitated by an experienced moderator and co-moderator using a semi-structured interview guide. Member-checking was undertaken after each question and at the completion of the focus group to ensure the researchers understood the participants accurately. At the end of each focus group, participants completed a brief demographic questionnaire. The focus groups were audio recorded and professionally transcribed verbatim.

### Data Analysis

Inductive content analysis was independently performed by three researchers using QSR NVivo 7. The researchers met after completing their individual analysis to compare and agree upon the identified themes that had emerged from the focus group data (as described by Miller & Crabtree) [25]. Strategies, as described by Guba and Lincoln [26], were used throughout the data collection and analysis processes to ensure the trustworthiness of the findings (see Table 1), and ethical approval for this study was obtained from the University of Western Ontario's Office of Research Ethics. Please see van Zandvoort et al. and Tucker et al. for a complete description of the study methods [19,20].

**Table 1:** Measures to Ensure Data Trustworthiness

Credibility	Member checking was conducted between each focus group question and at the end of each focus group to make certain that the researchers accurately understood the answers provided by participants.
Confirmability	Two researchers separately and concurrently performed inductive content analysis, and later met to compare their findings. We scrutinized data for similarities and differences across the interviews, and acknowledged emerging themes. The researchers discussed and prepared a summary of the analysis.

Dependability	Upon the completion of each focus group, two researchers met to debrief and summarize. Also, the researchers expressed any biases, which were then recorded and considered to ensure that the analyses were not partial to researcher bias. We documented focus group respondents' demographic information, and focus group location and participation rate for the purpose of an audit trail.

Transferability	We have explained the research process in detail, thus allowing interested researchers the ability to establish whether our results are transferable to their study and participants.

adapted from Irwin et al. 2005[23]; Tucker, Gilliland and Irwin 2007[27])

## Results

Participants discussed the impact of the home environment on physical activity behaviours of preschoolers and the themes that emerged have been captured under the following three themes which are discussed in detail below: 1. parents and the home environment as facilitators of physical activity; 2. parents and the home environment as barriers to physical activity; and, 3. suggestions for improving preschoolers' physical activity behaviours.

### Parents and the Home Environment as *Facilitators *to Physical Activity

Role Modelling. When asked about the facilitators to engaging preschoolers in physical activity while in childcare, in addition to items related to the childcare setting (see van Zandvoort et al.) [20], a number of participants underscored the essential role that parents play in providing opportunities for preschoolers to be physically active on a daily basis. For example, one childcare provider said, "we have a lot [of parents] that walk to pick them [their children] up and walk them home. We have a lot that will ride them on their bikes to school, ...bring them into childcare and then ride their bike home from childcare at the end of the day." Another echoed, "...we have a few parents that walk to school and home and I think that obviously encourages physical activity." One childcare provider also emphasized that parents who are physically active themselves tend to be "...out riding their bikes around, they're doing things with [their children] and it's because you don't [need] to have a lot of money to do things with your child." Childcare staff highlighted the importance of parental role modeling to encourage physical activity participation among young children and explained that simply playing outdoors is a cost-effective way to facilitate active play.

Enrolment in Extracurricular Activities. A few childcare providers also emphasized that many parents enrol their children in organized extra-curricular activities as a source of physical activity outside of childcare hours. One provider explained, "...they [the children] are so involved in soccer and swimming and hockey and all that in their evenings at home; our crowd is active almost every night in their personal lives...." When referring to the children's out-of-care activities, an additional provider listed, "...swimming lessons, the hockey, the skating, the soccer, and the baseball...." Though this provider went on to explain that not all children are able to participate in organized physical activities outside of childcare, "...[t]here is quite a few that do those extra activities but there [are] also the ones that can't afford it...." According to the childcare providers, many parents were supporting activity participation among their preschoolers by enrolling them in structured extracurricular sport and activities.

### Parents and the Home Environment as *Barriers *to Physical Activity

Lack of Encouragement for Physical Activity. When asked about the barriers to engaging preschoolers in physical activity, in addition to the items related to the childcare setting (see van Zandvoort et al.) [20], many childcare providers indicated that several parents did not seem to encourage an active lifestyle among their children outside of childcare hours. For example, one provider explained, "...when children go home on the weekends there is no outdoor or active play." Another elaborated, "...[i]t's a vicious cycle [and] you see it more and more...[i]f the parents are inactive the kids are." Yet another childcare provider stated, "I think there is definitely a decrease in just spur-of-the-moment...activities outside with your family. I don't think a lot of them do a lot of that kind of stuff...."

A number of the childcare providers spoke of their perceptions of the impact that inactive home-lives have on preschoolers' physical activity behaviours. One explained, "[on] Mondays they [the children] are very lethargic and [say] 'I don't want to. I'm bored.' By the end of the week you can see the influences of the [childcare providers]...and the playing they're doing [has] changed when they go home on the weekend. And [we] start all over again on Monday." Another provider said, "[b]ecause they don't do it [physical activity] at home...we hear 'oh I'm tired,' 'I can't do it,' [or] 'this is too much work.'"

Screen-viewing Behaviours. Some providers felt that even the children who were enrolled in extracurricular activities may not be physically active outside of the time spent at childcare or organized activities. One childcare provider expressed, "...when they're not at those [organized] activities all they do at home is play video games...it doesn't sound like they do any gross motor [activities] at home really." Another provider, lamenting on the challenge of time spent playing video games and watching television, said, "... the video games and the TV... they're overly subjected to that and finding entertainment and enjoyment out of physical activity sometimes isn't as exciting to them as the video games and the shows that they're watching." Another explained that children have become so enamoured by video games and television that they simply enjoy them more than they enjoy physical activity. She explained that she often hears from both parents and the kids in her care that "[TV/video is] the privilege they get taken away now, it's not like 'oh you can't go outside today, you didn't have a good day...' it's always like 'no video games, your computer is taken away, your movie or TV time is taken away.'"

### Suggestions for Improving Preschoolers' Physical Activity Behaviours

A number of childcare providers suggested that preschoolers' physical activity levels might be increased through efforts to enhance parents' awareness of--and involvement in--their children's physical activity.

Increasing Parent Awareness of the Benefits of Physical Activity. A few providers emphasized the importance of parental awareness about the benefits of physical activity and opportunities for activity in their neighbourhoods. For example, one said, "[j]ust like what you do with us as educators, like professional development for us...it's kind of like for the families; if you could help to promote it or inform them [parents] then maybe they might go [to neighbourhood activities and programs]...." Another provider shared, "[i]t's all about education. I mean people say 'oh, I don't need that,' [but] it's ignorance, it's lack of education. Any knowledge is good, is helpful, and I think it can stop that cycle [of inactivity] as far as being healthy and having a healthy family." As a possible way to deliver this information to parents, one provider suggested they "...do a newsletter for the area. For... 'at this arena this night is available and it's free'...because maybe they [parents] don't know and maybe sending something out might encourage knowing it's free...." A different provider shared, "[w]e've done different [homework] things with more academic [subjects]... like reading...We could maybe do something along those lines in way of physical activity... [for example, asking the children] 'did you do 5 minutes of skipping?'...." While it was suggested that workshops could be held at childcare centres to provide physical activity information to parents, one provider indicated (based on past experience) that this approach does not always target the parents in greatest need. The provider explained, "...a few years ago we did a workshop on media violence but again...you get the gung hoe parents who... want to know everything to make their child's life as optimal as it can be and the ones who need to be there are not because they don't want to come." This childcare provider further highlighted that even when free babysitting and food was provided during the workshop, often times parents didn't come.

Childcare Staff and Parental Partnerships to Support Physical Activity. A number of childcare providers also felt a valuable way to increase children's activity behaviours was to engage parents in activities with their children. For example, one provider emphasized, "...[t]he biggest part of parent involvement is they [parents] aren't role modeling and they don't want to be involved." By contrast, a couple of providers shared past positive experiences they had had with parents who participated in activities in their child's classroom, one of which shared, "...[w]e had a parent come in and they did an activ[ity]...about outer space with the children. The children had to move around and become a moving solar system. So getting parents involved... can also be another way of bringing that excitement in...They have ideas that we wouldn't necessarily think of." Another provider spoke about a game night that the children had requested, which was held at the childcare centre in the evening and offered children the opportunity to compete against their parents in a physical game. She explained, "...we had a phenomenal turnout [at that event]... [t]he parents all came in very much complaining, but the kids were hyped...once they [the parents] came back out they had such fun." While inviting parents into the childcare centre might not always be possible, it is clear that the partnership between childcare staff and parents is essential to facilitate healthy active behaviours among young children. One childcare provider highlighted the importance of open communication and complementary physical activity messaging when she said, "[i]f we don't educate the parents, how are we going to educate the kids. We need to work together with them. It's very important....[it's] like toilet training...you need to have the parents doing it with you. If not, it's not working."

## Discussion

The purpose of this study was to explore childcare providers' perspectives of the role of parents and the home environment on supporting physical activity behaviours of preschool-aged children enrolled in childcare centres. What was not expected, and therefore, was particularly enlightening, was the reliance of childcare staff on parents/guardians to create a home environment that complements the positive and encouraging physical activity messaging children may receive in childcare. This finding is important given that parents of preschoolers have previously acknowledged their dependence on childcare staff to ensure their children are sufficiently active [23,24]. Hearing childcare staff place a similar expectation on parents highlights the importance of parent-childcare staff partnerships that work together in service of developing healthy and active preschoolers. This finding warrants discussion because there has been increased attention placed on the childcare environment as a venue to support activity behaviours [28-32], and yet, staff are reporting that they cannot be fully successful in encouraging this important health behaviour if support and reinforcement for activity are lacking in the child's home. Physical activity is a complex behaviour that is influenced by a number of individuals (e.g., parents, childcare staff, siblings, etc.) and environments (e.g., home, childcare, and neighbourhood). Therefore, as mentioned above, it seems necessary that parents and childcare providers work together to ensure that preschoolers engage in sufficient physical activity. One plausible way to achieve this partnership might include activity documentation; if parents and childcare staff recorded the amount of activity children engaged in daily, this would serve to inform their care counterpart of the need for additional activity within their home or childcare environment. Having a better line of communication between the childcare staff and parents would allow these individuals to ensure their children, or the children they care for, are engaging in appropriate amounts of activity. Moreover, this communication will result in confirmation that their efforts are being continued and supported in the counter-environment (e.g., parents support of preschooler activity is extended to the childcare environment and vice versa). These partnerships might be maximized by providing educational material and resources for both parents and childcare staff to increase their confidence and comfort in supporting active behaviours among young children.

Childcare staff also noted the importance of parental role modeling to encourage active play among young children. Parents themselves have acknowledged the importance of their own role modeling in previous research [23]. Parental role modeling is often captured using parent activity levels [33]. Interestingly, in a systematic review of environmental correlates of physical activity in youth, Ferreira and colleagues noted that during childhood, fathers are more influential role models for children of both genders [34]. Specifically, fathers' physical activity levels appear to be associated with physical activity levels of children despite genders, while mothers' activity levels appear to be linked with girls' activity behaviours [34]. Among preschoolers, Sallis and colleagues identified that parental physical activity levels were significantly correlated with child activity participation and suggested that the impact of parental role modeling may extend to venues outside the home [15]. This suggests the influence and reach that parental role model plays in fostering health behaviours among their children. Parental support for physical activity has been identified as a significant positive influence on children's physical activity participation [35]; that is, by playing with children, providing transportation to parks and activity facilities, and reinforcing physical activity participation, parents can increase physical activity participation among preschoolers [35]. In fact, Oliver and associates recently suggested that increased preschooler physical activity is a result of parent activity prioritization and parental support [36], and Moore and colleagues noted that preschoolers with two active parents were 5.8 times more likely to be active than preschoolers with inactive parents [16]. Clearly, encouragement of appropriate physical activity levels among young children is crucial in the home environment, and mechanisms to support parents to engage in activity with their children are necessary.

An additional finding of this study was the providers' perspectives that children spend a substantial amount of time in sedentary behaviours at home. While the results of this study do not come from parents, children often tell their childcare provider(s) what they did on the weekend, and participants reported that many of their charges spend a great deal of time in front of a screen (e.g., television, computer, video game). This findings is not surprising given the national average for screen time among Canadian youth is 6 hours per day on weekdays and 7 hours per day on weekend days, and that 27% of 2-3 year old toddlers and 23% of 4-5 year old preschoolers engage in more than 2 hours of screen time per day [7]. Parents of preschoolers have previously reported that their children spend 1 to 5 hours a day in front of a screen and that the television is often used as a "babysitter" [37]. With the increased presence of both parents in the workforce, parents of preschoolers have reported that they are "too busy" and "too tired" to actively play with their children [38], resulting in increased time in sedentary behaviours. The high rate of screen viewing, and consequently, sedentary behaviours, is problematic given the rising rates of childhood obesity, and efforts are needed to entice families to turn off the screens and engage in active play together.

Although the present study provides a number of important findings, the limitations of this research must be acknowledged. First, it must be noted that because the intended primary focus of this research was not the home environment, and instead it was an unanticipated theme about which caregivers focussed, we may have missed asking some focus group questions that probed even deeper into this area. Secondly, the participants included a convenience sample of very eager childcare providers and the results of this study are limited to our sample. Regardless of these limitations, the present study offers valuable insights into the need for parent-caregiver partnerships and the importance of providing a supportive home environment with parents who actively play with their children.

## Conclusion

The current study provides insightful and unique information regarding childcare providers' perspectives about the importance of the home environment in supporting preschoolers' physical activity participation. The results of this study highlight the need for parents and childcare staff to work together to engage preschoolers in ample activity and to communicate similar messaging regarding the importance of this health behaviour. Specifically, the examples provided by participants highlight the need for effective staff and parent partnerships in service of developing active behaviours among young children; in other words, the effort and education that transpires in the childcare centre could be lost if children go home to an environment and parents that do not support or value physical activity. As discussed above, previous research has found that physical activity participation among preschoolers is linked to parents' physical activity levels (role modeling) and an activity-supporting home environment [35]. In the current research, participants acknowledged the opportunity and necessity for increased collaboration and partnership between childcare providers and parents to support healthy active behaviours among these young citizens.

## Competing interests

The authors declare that they have no competing interests.

## Authors' contributions

PT conceived of the study and oversaw all aspects. PT also completed a number of the focus groups, was responsible for data analysis and drafting the manuscript. MvZ completed data collection and analysis and assisted with the first draft of the manuscript. SB assisted with data analysis and provided feedback on the manuscript. JI assisted with data collection and provided feedback on the manuscript. All authors contributed to the design of the study and read and approved of the final manuscript.

## Pre-publication history

The pre-publication history for this paper can be accessed here:

http://www.biomedcentral.com/1471-2458/11/168/prepub
